# Supplementary material for: Rift Valley Fever Virus Exposure amongst Farmers, Farm Workers, and Veterinary Professionals in Central South Africa
Source: Viruses. 2019 Feb 7;11(2):140. doi: 10.3390/v11020140 (PMC6409972; doi:10.3390/v11020140)
Supplement: Supplementary file 1 [file viruses-11-00140-s001.pdf]

Table S1. Input distributions and parameter for Monte Carlo simulations of case projection.

Input distribution		Parameters	
$\boldsymbol{TP \sim Beta(\alpha, \beta)}$	$\alpha = 54$ (successes/seropositives)	$\beta = 597 - 54$ (failures/seronegatives)[1].	
$\boldsymbol{No. of\ LOHH\ \sim Pert(a, b, c)}$	$a = 4728$	$b = \frac{(4728+8089)}{2}$	$c = 8089$
$\boldsymbol{No. of\ employees\ per\ LOHH\ \sim Normal(\mu, \sigma)}$	$\mu = \frac{1265}{192}$ (number of employees/number of farms sampled)	$\sigma = \frac{6.58806}{\sqrt{192}}$ (standard error of mean)	

[1] The survey found 62 seropositives and (685 – 62) seronegatives; however, α and β were reduced in order to give the same seroprevalence but reflect the reduction in precision for cluster vs. simple random sampling.

Minimum	1849
Maximum	6781
Mean	3849.435
Median	3801
Mode	3383
Left X	2792
Left P	5%
Right X	5092
Right P	95%

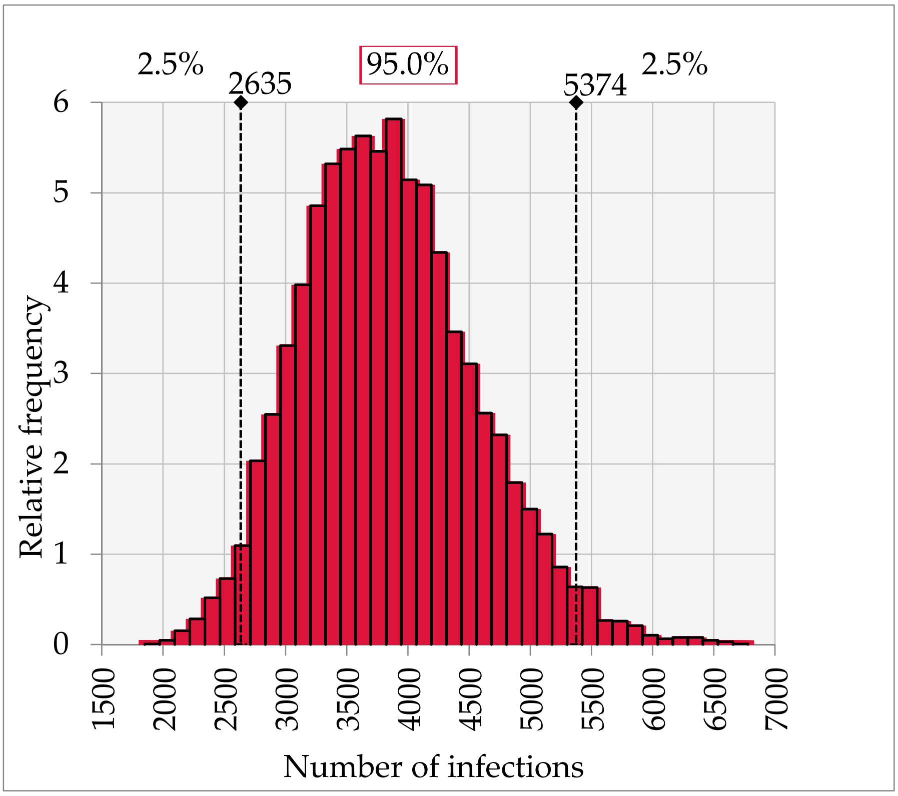

Figure S1. Distribution of possible outcome values of historically infected RVF cases in the study area.

Table S2. Table Demographic distribution of participants and farm size distribution

Study group	Farmers and workers	Veterinary professionals
Job category	Workers/herdsman:487 (71%)<br>Farmers/livestock owner/manager: 173 (25%)<br>Family/domestic worker/driver: 24 (4%)	Veterinarian: 66 (54%)<br>Veterinary technician/animal health technician/paraveterinarian: 37 (30%)<br>Veterinary nurses: 9 (7%)<br>Researchers: 5 (4%)<br>Game/nature conservators: 3 (3%)<br>Veterinary professionals who practiced farming: 2 (2%)
Sex distribution	Male: 634 (93%)<br>Female: 50 (7%)	Male: 68 (49%)<br>Female: 70 (51%)
Age distribution	16-29 yr: 196 (30%)<br>30-39 yr:196 (30%)<br>40-49 yr: 114 (18%)<br>50-63 yr: 106 (16%)<br>≥64 yr: 40 (6%)<br>Median: 36 (interquartile range (IQR: 28-48)	16-29 yr: 28 (22%)<br>30-39 yr: 50 (39%)<br>40-49 yr: 20 (16%)<br>50-63 yr: 22 (17%)<br>≥64 yr: 7 (6%)<br>Median: 37 (IQR: 30-48)
Private farm size distribution	0-20 ha: 11 (6%)<br>21-100 ha: 9 (5%)	

	101-500 ha: 26 (14%)<br>501-1000 ha: 33 (18%)<br>1001-2000 ha: 27 (14%)<br>2001-5000 ha: 52 (28%)<br>5001-10000 ha: 20 (11%)<br>10001-max ha: 7 (4%)<br>Median: 1001-2000 ha
Communal land use size distribution	0-20 ha: 3 (16%)<br>21-100 ha: 3 (16%)<br>101-500 ha: 4 (21%)<br>501-1000 ha: 6 (32%)<br>1001-2000 ha: 1 (5%)<br>2001-5000 ha: 2 (10%)<br>Median: 393 or 101-500 ha
